# Supplementary material for: The Ontology of Biological Attributes (OBA)—computational traits for the life sciences
Source: Mamm Genome. 2023 Apr 19;34(3):364–78. doi: 10.1007/s00335-023-09992-1 (PMC10382347; doi:10.1007/s00335-023-09992-1)
Supplement: Supplementary file 1 — Supplementary file1 (DOCX 13 kb) [file 335_2023_9992_MOESM1_ESM.docx]

## Supplementary Materials

**S1: All DOS-DP patterns used by OBA**

| **Pattern name** | **Description** | **Example** |
| --- | --- | --- |
| attribute_location | This pattern describes an attribute of a specified entity that can be observed, measured or quantified in a specific location. | OBA:0000091 lysosomal lumen pH |
| chemical_role_attribute | This pattern describes an attribute of a chemical entity, where the chemical entity is a category of chemicals with a specific role. | OBA:0000079 hormone levels |
| chemical_role_attribute_location | This pattern describes an attribute of a chemical entity in a specified location, where the chemical entity is a category of chemicals with a specific role. | OBA:VT0003369 blood estrogen amount |
| disease age of onset | Age at which disease manifestations first appear. | OBA:2001000 age of onset of Alzheimer disease |
| entity_attribute | This pattern describes an attribute of a specific entity that can be observed, measured or quantified. | OBA:0002360 trochanter size |
| entity_attribute_location | This pattern describes an attribute of a specific entity that can be observed, measured or quantified in a specified location. | OBA:2020005 lysine in blood amount |
| entity_attribute_part_of | This pattern describes very general grouping traits. This should be used only in conjunction with morphology and quality attributes. | OBA:1000013 cytoskeleton morphology |
| process_attribute_location | This pattern describes an attribute of a biological process in some location. | OBA:2050069 serum lipase activity amount |
| response_to_chemical_stimulus_trait | This pattern describes a trait that affects the biological response to a chemical. | OBA:2040012 trait in response to morphine |
| response_to_chemical_with_role_stimulus | This pattern describes a trait that affects the biological response to a chemical, where the chemical is a category of chemicals with a specified role. | OBA:2040009 trait in response to endocrine disruptor |

**S2: Supplementary query (SPARQL): Aggregate all data related to the morphology of the a part of the cardiovascular system**

| Note: This query works when *part_of (BFO:0000050)* and *subclass_of (rdfs:subclassOf)* edges are materialised, like in Ubergraph (<https://ubergraph.apps.renci.org/sparql>). Otherwise, wildcard operators (*) need to be added to the properties. |
| --- |
| PREFIX subclass_of: <http://www.w3.org/2000/01/rdf-schema#subClassOf>  PREFIX label: <http://www.w3.org/2000/01/rdf-schema#label>  PREFIX characteristic_of: <http://purl.obolibrary.org/obo/RO_0000052>  PREFIX part_of: <http://purl.obolibrary.org/obo/BFO_0000050>  PREFIX biological_attribute: <http://purl.obolibrary.org/obo/OBA_0000001>  PREFIX morphology: <http://purl.obolibrary.org/obo/PATO_0000051>  PREFIX cardiovascular_system: <http://purl.obolibrary.org/obo/UBERON_0004535>  PREFIX tissue: <http://purl.obolibrary.org/obo/UBERON_0000479>  SELECT DISTINCT ?oba ?oba_label ?entity_label ?anatomy ?anatomy_label  WHERE {  ?oba subclass_of: biological_attribute: .  ?oba subclass_of: morphology: .  ?oba characteristic_of: ?entity .  ?entity part_of: cardiovascular_system: .  ?entity subclass_of: tissue: .    ?entity label: ?entity_label .  ?oba label: ?oba_label .  } |
